# Impact of the COVID-19 pandemic on cataract surgeries in Brazil: A retrospective study

**DOI:** 10.1016/j.clinsp.2024.100380

**Published:** 2024-05-15

**Authors:** Silvana Rossi, Rafael Scherer, Priscilla Jorge, Newton Kara-Junior

**Affiliations:** Department of Ophthalmology, Faculdade de Medicina da Universidade de São Paulo (FMUSP), São Paulo, SP, Brazil

**Keywords:** Cataract surgery, Epidemiology, Prevention of blindness, Pandemic, Covid-19

## Abstract

**Purpose:**

This study aimed to describe the behavioral patterns of data on cataract surgery performed in the Brazilian public health system before, during, and after the COVID-19 outbreak and estimate the setbacks generated by the pandemic to guide public policies.

**Materials and methods:**

This was an observational, longitudinal, and descriptive epidemiological study based on data retrieved from the public health system's databases of cataract surgeries performed each year and in each region from 2015 to 2022.

**Result:**

In Brazil, compared with the average of the 5-years preceding the pandemic, a 23 % reduction in the number of cataract surgeries was observed in 2020, followed by a 21 % increase in 2021, compensating for the majority of patients that were not operated on. However, the worsening situation of blindness caused by cataracts due to the pandemic not be avoided in the Central-West region, where unrecovered cases continue to accumulate.

**Conclusion:**

The COVID-19 pandemic did not worsen the situation of cataract blindness in Brazil due to the efficacy of the measures taken by the government in resuming elective surgeries. However, the auhtors recommend that the distribution of resources for cataract surgeries should consider regional discrepancies based on epidemiological data.

## Highlights

• When regional data were analyzed, it became clear that the worsening of blindness due to cataracts as a result of the pandemic was not avoided in the midwest region.
• The assessment took place during a critical period of the COVID-19 pandemic; thus, the present study contributes to these findings and suggests that new epidemiological studies be carried out to monitor this worsening.

## Introduction

Due to the COVID-19 pandemic, a public health emergency was declared in Brazil in 2020 through Ordinance MS/GM n° 188 of February 3, 2020.^1^ During this period, the demand for hospitalization of patients with COVID-19 increased, which resulted in a reduction in the number of elective cases.^2^^,^^3^ By overloading the health network, the pandemic has affected treatment for other diseases, including cataract surgery, the effects of which may last, particularly if regional disparities are considered as complicating factors.

In the period of 2020–2021, the most critical time of the COVID-19 pandemic, there was a redirection of care and procedures throughout the healthcare sector.^1^^,^^2^^,^^4^ In Brazil, the public health system, which offers free medical coverage to 75 % of the population, was overwhelmed,^3^ and there was a shortage of doctors because many professionals fell ill or were transferred to the front line of the fight against COVID-19.^5^^,^^6^

In ophthalmology, cataract surgery, one of the most commonly performed surgical modalities, was hampered due to the pandemic.^7^^,^^8^ Because cataracts are among the main causes of blindness not only in Brazil but also worldwide,^8^, ^9^, ^10^ it is necessary to estimate the magnitude of the reduction in surgical supplies during the pandemic to identify the problem and guide the development of public policies toward its solution.

Therefore, this study aimed to estimate the setback induced by the pandemic in the performance of cataract surgeries in the public health system (SUS) in Brazil and its regions. This would help measure the magnitude of the problem that the government needs to overcome to mitigate the indirect effects of the pandemic on the prevalence of blindness caused by cataracts.

## Materials and methods

An observational, longitudinal, and descriptive epidemiological study was conducted covering the following databases: DATASUS, Ministry of Health, Brazilian Institute of Geography and Statistics (IBGE), Portal Fundo de Saúde, and Portal da Transparência. The study was previously submitted for ethical consideration and approved (#50,811,221.2.0000.0068). Data related to outpatient production (SIA/SUS) and hospital production (SIH/SUS) were collected on December 8, 2022 and reviewed on April 10, 2023.^6^^,^^11^ Using TABNET, a public domain tabulator developed by DATASUS,^11^ data on the quantities and costs of cataract procedures performed in hospitals or outpatient clinics by the SUS in Brazil from 2015 to 2022 were obtained by year and region. The studied procedures were extracapsular cataract extraction with (code 405,050,097) and without (code 405,050,100) Intraocular Lens (IOL) implantation and phacoemulsification with rigid IOL implantation (code 405,050,119) and with foldable IOL (code 405,050,372). The average number of cataract surgeries performed by the public health system in Brazil in the 5-years preceding the pandemic was compared with the years 2020, 2021, and 2022.^9^^,^^11^, ^12^, ^13^, ^14^

From the Transparency Portal[6] and Health Fund Portal,^7^ data on expenditure on cataract surgeries and healthcare in 2021 and 2022 were obtained by region. Population estimates for each region were also obtained from IBGE. These data were used to calculate health expenditure per capita and cataract surgery expenditure per inhabitant in 2021 and 2022.

## Results

The present results revealed that due to the pandemic; 35,344 individuals in the Central-West region who became blind because of cataracts remain unoperated due to the pandemic. However, in the North region, from 2020 to 2022, 63,558 more surgeries were performed than necessary to compensate for the patients who had not been operated on due to the pandemic (Table 1).

**Table 1:** The number of cataract surgeries performed from 2015 to 2022 by the Public Health System in Brazil and by region, as well as the percentage variation from 2020 to 2022 and the estimated cumulative demand in the period, in comparison to the average of the 5-years preceding the pandemic.

		Region				
Year	Brazil	Southeast	Northeast	South	Midwest	North
2015	473.585	207.016	123.959	73.748	39.189	29.673
2016	456.297	180.305	131.075	69.554	45.688	29.675
2017	490.015	207.769	130.635	77.559	42.775	31.277
2018	563.879	225.937	156.529	101.797	47.023	32.593
2019	662.536	277.084	200.685	119.864	31.277	33.626
**5-year average**	**529.262**	**219.622**	**148.577**	**88.504**	**41.190**	**31.369**
2020	406.558 (−23.18 %)^a^	173.929 (−20.80 %)^a^	110.477 (−25.56 %)^a^	68.480 (−22.63 %)^a^	18.146 (−55.98 %)^a^	35.526 (13.23 %)^a^
2021	640.408 (21.00 %)^a^	267.860 (22.00 %)^a^	186.549 (25.54 %)^a^	95.027 (7.37 %)^a^	27.293 (−33.72 %)^a^	63.679 (131.00 %)^a^
2022	862.713 (62.98 %)^a^	357.026 (62.50 %)^a^	279.991 (88.40 %)^a^	124.449 (40.60 %)^a^	42.787 (3.90 %)^a^	58.460 (86.30 %)^a^
Accumulated demand at the end of 2021	−11.558	‒	−128	−13.501	−36.941	‒
Accumulated demand at the end of 2022	‒	‒	‒	‒	−35.344	‒

a Percentage change from the five-year average leading up to the pandemic.

**Fig. 1:**
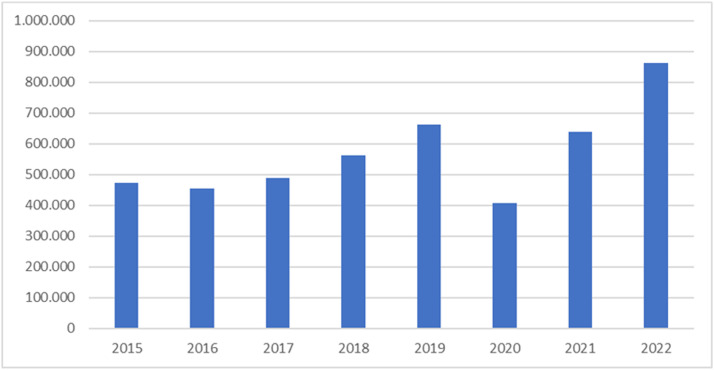
The number of cataract surgeries performed from 2015 to 2022, in public health system in Brazil.

**Fig. 2:**
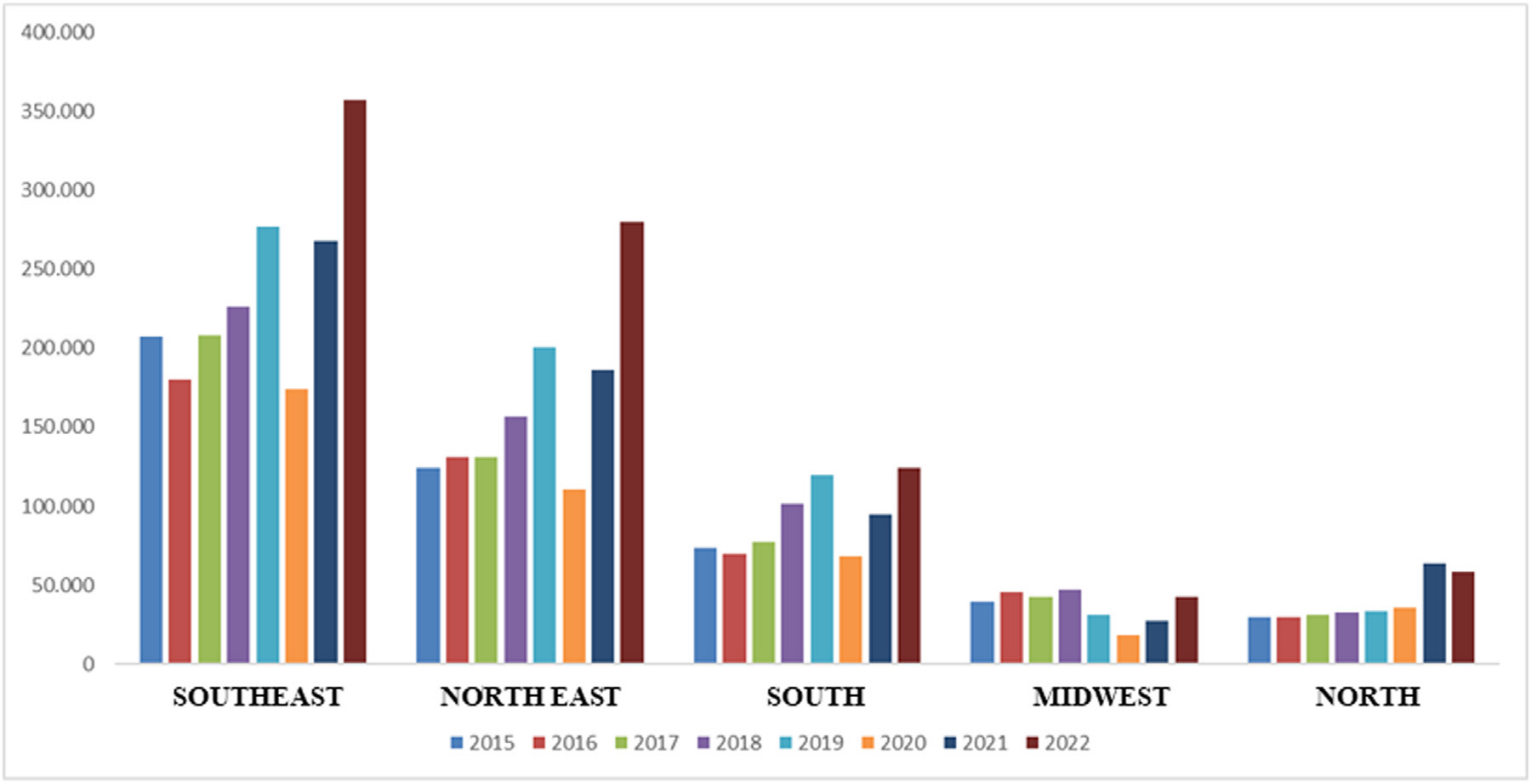
The number of cataract surgeries performed from 2015 to 2022, by the public health system in Brazil, separated by region.

From 2016 to 2019, the number of surgeries performed showed an increasing trend (Fig. 1, Fig. 2). In 2020, the start of the pandemic, the number of cataract surgeries decreased, but after that year, there was a more significant increase in the number than in 2019, the 5-years preceding the pandemic.

The same increasing trend between 2016 and 2019 was observed in the Southeast, Northeast, and South regions but not in the Central-West region. In the North region, the number of surgeries during the pandemic decreased. In the South region, the post-pandemic recovery was not as pronounced as in other regions.

In 2021, the per capita government funding for the health sector was lower in the North and Central-West regions than in the other regions (Table 2). However, based on the distribution of resources, the North region directed a large portion of its budget to investments in cataract surgeries (0.56 %), whereas in the Central-West region, investment in this type of procedure was low (0.28 %).

**Table 2:** Government spending on health and cataract surgery in 2021, by region and per inhabitant.

Regions	Health expenditure	Per capita expenditure on health^a^	Spending on cataract surgery	Spending on cataract surgery Inhabitant^a^
Southeast	R$ 37,7 billion	R$ 420,60	R$ 204 million	R$ 2,28
Northeast	R$ 28,89 billion	R$ 500,97	R$ 144 million	R$ 2,49
South	R$ 15,00 billion	R$ 493,37	R$ 73 million	R$ 2,41
Midwest	R$ 7,63 billion	R$ 456,68	R$ 21 million	R$ 1,26
North	R$ 8,55 billion	R$ 452,42	R$ 48 million	R$ 2,56

Source: Transparency Portal/Localiza SUS/Federal Senate. Data up to January 15, 2021. https://www.gov.br/pt-br/noticias/financas-impostos-e-gestao-publica/2021.

a Population data estimated by Brazilian Institute of Geography and Statistics.

In 2022, the Northeast and Southeast regions invested the most in cataract surgeries (0.72 % and 0.71 %, respectively), whereas the Central-West region invested the least (0.43 %) (Table 3). In 2021, expenditure on cataract surgeries in the Southeast, Northeast, South, and Central-West regions increased, whereas that in the North region decreased.

**Table 3:** Government expenditure on health and cataract surgery in 2022, by region and per inhabitant.

Regions	Health expenditure	Per capita expenditure on health^a^	Spending on cataract surgery	Spending on cataract surgery Inhabitant^a^
Southeast	R$ 38,19 billion	R$ 447,07	271 million	3.03
Northeast	R$ 31,3 billion	R$ 568,66	225 million	3.90
South	R$ 14,97 billion	R$ 493,01	95 million	3.14
Midwest	R$ 7,76 billion	R$ 467,16	33 million	1.95
North	R$ 8,82 billion	R$ 529,17	44 million	2.31

Source: Transparency Portal/Localiza SUS/Federal Senate. Data up to January 15, 2021. https://www.gov.br/pt-br/noticias/financas-impostos-e-gestao-publica/2021.

a Population data estimated by Brazilian Institute of Geography and Statistics.

## Discussion

According to the present study, the number of cataract surgeries decreased by 23 % in 2020 compared with the average of the previous 5 years. The main causes for this phenomenon are believed to be directly or indirectly related to the pandemic, such as the suspension of elective surgeries from March to June 2020, restrictions on the movement of people, and people's fear of leaving home.^11^^,^^14^

In Brazil, in 2021, even without an epidemiological analysis of the extent of the damage caused by the pandemic to the population's visual health, measures were implemented to increase the number of cataract surgeries in the public health system, such as public-private partnerships with social organizations; the Corujão da Saúde Project, in which procedures were performed at night and on weekends, taking advantage of idle installed capacity; or the Strategic Actions and Compensation Fund (FAEC) .^2^^,^^15^

Thus, with the start of vaccination and the easing of pandemic restrictions in 2021, the number of cataract surgeries increased by 21 % compared with the average in the period before the pandemic. Such an increase was sufficient to compensate for the majority of patients who were not operated on in the previous year. It was observed that by the end of 2021, only around 11,558 individuals remained blind from cataracts in Brazil as a result of the COVID-19 pandemic.

In 2022, the number of cataract surgeries performed increased by 63 % compared with the average in the period before the pandemic, not only fully compensating for the demand accumulated due to the pandemic but also contributing to the improvement in the situation of blindness due to cataracts in Brazil.^6^^,^^7^^,^^14^

However, regional disparities were identified as complicating factors in Brazil. In 2021, while the Southeast and Northeast regions followed the national variation and practically treated all patients that had not been operated on in the previous year, the South region did not show recovery in the number of surgeries observed in the rest of the country and showed around 13.500 individuals still with cataract, people who could have already been rehabilitated if it were not for the pandemic. Only in 2022, the South region managed to offset the accumulated demand.

However, the biggest contrast was in the Central-West and North regions. While the former showed the biggest drop in the number of cataract surgeries performed in 2020 and was the only one that did not show recovery in 2021, limiting itself to the pre-pandemic levels in 2022, the latter behaved as if the pandemic had not existed, being the only one that managed to maintain the surgical rate in 2020 and practically double the number of surgeries in the following years, reaching record rates during the studied period.

The authors believe that government resources needed to meet demands arising from the pandemic have not been homogeneously distributed across the regions because the study estimated that 35,344 individuals were still blind in the Central-West region because of the pandemic. Furthermore, in the North region, from 2020 to 2022, 63,558 more surgeries than necessary were performed to compensate for patients who had not been operated on due to the pandemic.

In 2021, the per capita government funding for the health sector was lower in the North and Central-West regions than in the other regions. However, the North region directed a large portion of its budget to investments in cataract surgeries, whereas in the Central-West region, investment in this type of procedure was low. In 2022, the Northeast and Southeast regions invested the most in cataract surgeries, whereas the Central-West region invested the least. Expenditure on cataract surgeries in the Southeast, Northeast, South, and Central-West regions increased in 2021, whereas that in the North region decreased.

This study shows that there were asymmetries in the distribution of health resources in different regions of the country. The authors believe that public health decisions should be taken based on the results of epidemiological studies to avoid harm to the population. Thus, it is necessary to investigate the real reasons and priorities that led to the targeting of health resources in the different regions of the country.

The data used in the present study were the most reliable data available to the public, as they were obtained and published by official institutions. Nevertheless, the authors depend on the reliability of these databases, which can be a source of bias and a limitation of this study.

## Conclusions

In the present study, the overall scenario of cataracts was evaluated in Brazil, where it was estimated that no people would remain blind due to cataracts in 2022, despite the limitations imposed by the COVID-19 pandemic on elective surgeries. The present results revealed that the actions implemented to reestablish elective surgeries in Brazil were effective. However, analysis of regional data revealed that the worsening situation of blindness due to cataracts during the pandemic was not avoided in the Central-West region, where cases continued to accumulate. This finding suggests the need to allocate government resources similar to those allocated in other regions, particularly in the North, to address this specific situation in the Central-West region.

## Authors’ contributions

Study design and planning: SR, NKJ. Data collection, analysis, and interpretation: SR, NKJ. Manuscript drafting or review: SR, RS, PJ, NKJ. Approval of the final version: SR, RS, PJ, NKJ. Public responsibility for the content of the article: SR, RS, PJ, NKJ.

## Declaration of competing interest

The authors declare no conflicts of interest.
